# Risk Factors for Postoperative Complications and In-Hospital Mortality Following Surgery for Cervical Spinal Cord Injury

**DOI:** 10.7759/cureus.31960

**Published:** 2022-11-28

**Authors:** Alexander Wilton

**Affiliations:** 1 Orthopaedics, Northern Sydney Local Health District, Sydney, AUS

**Keywords:** australia, inhospital mortality, early postoperative complication, logistic regression model, cervical spinal cord injury, spinal cord injury

## Abstract

Background and objective

The operative priority in the setting of traumatic cervical spinal cord injury (SCI) is to decompress the injured spinal cord and stabilize the vertebral column. Currently, there is a relative paucity of evidence regarding associations of patient and surgical factors with in-hospital mortality following traumatic SCI. In light of this, the aim of this study was to investigate the correlation of injury, patient, and surgical factors with in-hospital morbidity and mortality.

Methods

The study was designed as a retrospective cohort study. The electronic medical records (EMR) at a single tertiary centre in Australia were retrospectively reviewed over a five-year period (2016-2021). All adults who were admitted to undergo emergency surgery for cervical SCI were identified and reviewed for patient factors (age, sex, comorbidities), injury factors [injury severity score (ISS), American Spinal Cord Injury Association (ASIA) classification], and surgical factors (anterior/posterior/360 instrumentation, greater than five levels instrumented, operative time). Factors were correlated to in-hospital complications (infection, pressure injury, ventilator dependency, venous thromboembolism, stroke) and in-hospital mortality by using univariate analysis and multivariable logistic regression models.

Results

A total of 92 patients were identified from the EMR. The median patient age was 54.5 years [interquartile range (IQR): 2.5]; 77 (82.2%) of the participants were male. The median ASIA classification was C4 ASIA C. In-hospital mortality following surgery was 6.5% (n=6). Of these patients, the primary cause of death was respiratory failure in 83.3% (n=5). In-hospital mortality was associated with anticoagulation (p=0.01), coronary disease (p=0.012), complete injury (p=0.011), and ventilator dependency (p<0.001). Postoperative pneumonia was associated with complete injury (p=0.009) and polytrauma (p=0.002). Ventilator dependency was associated with complete injuries (p<0.001) and polytrauma (p<0.001). A logistic regression analysis found complete neurological injury to be significant in predicting in-hospital mortality [odds ratio (OR): 184.53, 95% confidence interval (CI): 2.41-14106.65, p=0.018, R^2^=0.58].

Conclusion

To improve surgical outcomes in patients with traumatic cervical SCI, a concerted effort must be made to prevent postoperative complications. Cardiovascular comorbidities present significant risk factors for patients. Patient age appears to insignificantly influence postoperative complication rates; however, this finding may have been influenced by selection bias. Postoperative respiratory complications, especially in patients with complete neurological deficits, can be particularly devastating.

## Introduction

Acute cervical spinal cord injury (SCI) is a devastating injury that requires highly complex and sub-specialized multidisciplinary care. The estimated incidence of traumatic SCI in Australia is 21.0-32.3 per million population [1]. The rates are highest in young adult males, with the majority of injuries caused by accidental trauma [2,3]. The effects of surgery are both immediate and long-lasting. Decompression of the spinal cord may prevent the neurotoxic biochemical cascade of secondary injury mechanisms, while stabilization prevents the morbidity associated with chronic deformity [4-7].

Postoperative complications following surgery for cervical SCI can contribute to early mortality, affect neurological recovery and functional outcomes, and result in high healthcare costs [8]. Respiratory complication following cervical SCI is the main cause of in-hospital mortality, responsible for 42-87% of deaths [9-11]. Specialized respiratory care for cervical SCI patients can improve outcomes [12]. Increased risk of mortality has been associated with the development of pulmonary embolism (PE), which is most likely to occur in the immediate post-injury period [13]. Pressure ulcers (PU) are the most common complication of SCI and a major cause of chronic re-hospitalisation [14-16]. Currently, the levels of evidence about risk factors for the development of PU in the acute period remain insufficient [16].

Patient factors including age and comorbidities can influence the risk profile of SCI patients [9,10,17-19]. Complications in elderly patients are more likely to be cardiorespiratory in origin [myocardial infarction, deep vein thrombosis (DVT), PE] whereas infections (pneumonia, respiratory and urinary infection) are more frequent in younger groups of patients [10,17]. Studies have demonstrated a strong association between complete SCI and increased in-hospital mortality [9,11,19-21]. In-hospital mortality for patients aged over 65 years with complete cervical SCI is five times higher than those with incomplete injuries [19]. The severity of polytrauma has also been significantly associated with postoperative in-hospital mortality among SCI patients [18,21].

Surgical factors also influence complication rates. Existing evidence supports improved neurological recovery among cervical SCI patients undergoing early surgery [22], as well as lower in-hospital mortality [9]. A growing body of evidence supports early surgery as a means to reduce the time spent in intensive care and the overall hospital length of stay [23]. Medical complications such as PE may also be reduced by the early mobilization achieved by surgery [23]. Longer surgical duration is associated with increased postoperative complications [24]; however, anterior and posterior approaches may be equivalent in terms of outcomes in the long term [25]. Posterior approaches may result in greater blood loss, longer operative times, and longer inpatient admission duration [26].

There is a paucity of evidence regarding the association of these factors with in-hospital mortality and postoperative complications from an Australian cohort. Hence, this study aimed to investigate these associations by using a predictive model.

## Materials and methods

**Figure 1:**
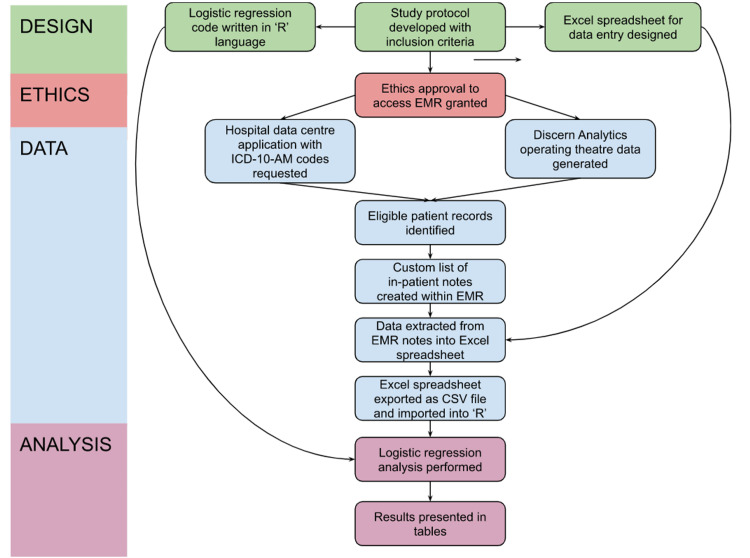
Flowchart depicting the study procedure EMR: electronic medical records; ICD-10-AM: the International Statistical Classification of Diseases and Related Health Problems, Tenth Revision, Australian Modification

An overview of the study procedure is outlined in Figure 1.

Ethical approval

The authority to access patient data through electronic medical records (EMR) was granted by the Northern Sydney Local Health District (NSLHD) Hospital Research Ethics Committee (HREC).

Study population

The Royal North Shore Hospital (RNSH) Spinal Cord Injury Service is one of two hospitals in the state of New South Wales that provides acute surgical and rehabilitation services for acute traumatic SCI patients. The RNSH caters to roughly half the population of NSW, which has a total population of 8.1 million people [27]. Adult patients over 16 years of age who sustained acute, traumatic cervical SCI from June 1, 2016, to June 1, 2021, and underwent operative management at RNSH under the orthopaedics team were eligible for inclusion in the study. The inclusion and exclusion criteria are outlined in Table 1.

**Table 1:** Patient inclusion and exclusion criteria RNSH: The Royal North Shore Hospital; SCI: spinal cord injury

Inclusion criteria	Exclusion criteria
Greater than 16 years of age	Subacute presentation (>48 hours after injury)
Acute traumatic SCI (injury within 48 hours before presentation)	Cervical SCI of aetiology other than trauma (i.e., malignancy, infection, ischemia, complications of medical or surgical intervention, periprosthetic fracture, or hardware failure)
Cervical decompression +/- fusion +/- fixation +/- bone grafting performed	Patients managed nonoperatively
Admission to RNSH under the orthopaedics team	Patients who died before surgery
	Patients managed with halo external fixation

Data collection

The hospital coding centre was approached with a request for data on admissions during the study period with unique billing codes. These codes were chosen from the International Statistical Classification of Diseases and Related Health Problems, Tenth Revision, Australian Modification (ICD-10-AM) [28] and are listed in Table 2.

**Table 2:** ICD-10-AM codes provided by the hospital data centre for patient search ICD-10-AM: the International Statistical Classification of Diseases and Related Health Problems, Tenth Revision, Australian Modification

ICD-10-AM code	Code description
S14.0	Concussion and oedema of cervical spinal cord
S14.10	Unspecified injury of cervical spinal cord
S14.11	Complete lesion of cervical spinal cord
S14.12	Central cord syndrome (incomplete cord injury) of the cervical spinal cord
S14.13	Other incomplete cord syndrome of cervical spinal cord
S14.70	Function spinal cord injury, cervical level unspecified
S14.71	Function spinal cord injury, C1
S14.72	Function spinal cord injury, C2
S14.73	Function spinal cord injury, C3
S14.74	Function spinal cord injury, C4
S14.75	Function spinal cord injury, C5
S14.76	Function spinal cord injury, C6
S14.77	Function spinal cord injury, C7
S14.78	Function spinal cord injury, C8

**Figure 2:**
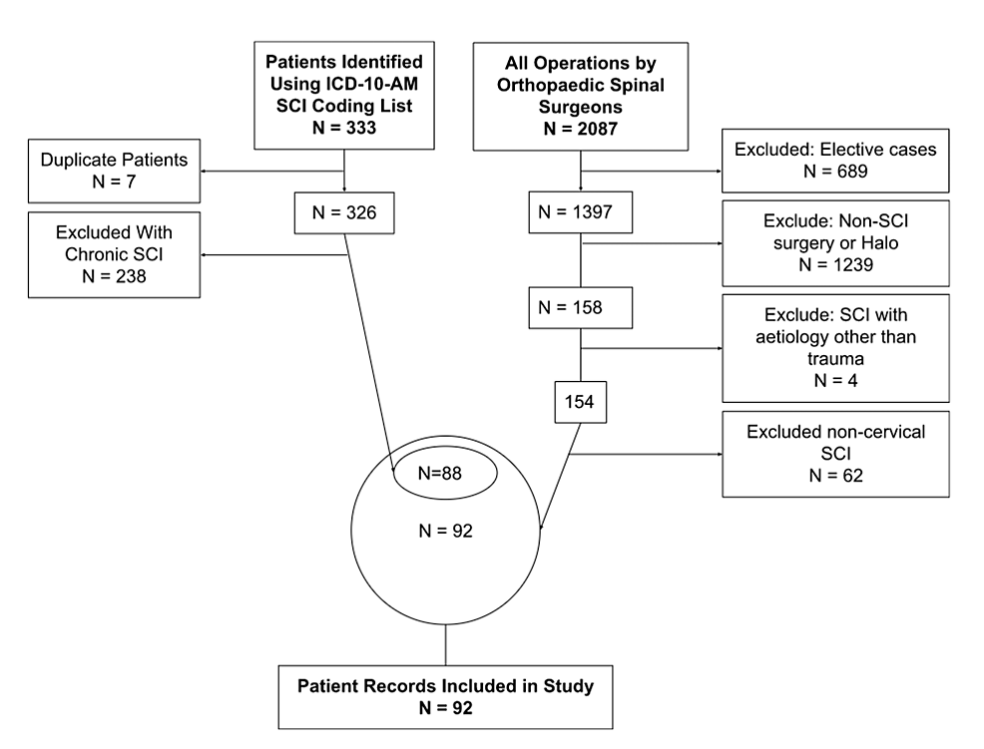
Flow diagram of sample selection ICD-10-AM: the International Statistical Classification of Diseases and Related Health Problems, Tenth Revision, Australian Modification; SCI: spinal cord injury

This process identified 88 patients who fulfilled the inclusion criteria (Figure 2). Additionally, a manual search of all emergency operations documented in the operating theatre EMR was performed using the data collection tool, Discern Analytics 2.0 (DA2) within Surginet (Oracle Cerner, 2016, Austin, TX), which yielded an additional four patients missed by hospital coding. The triage note, admission notes, operation reports, and medical imaging were reviewed and relevant data were extracted to a pre-designed Microsoft Excel spreadsheet​​ ​​(Microsoft Corporation, Redmond, WA).

Patient age, sex, and comorbidities including smoking, hypertension, diabetes, anticoagulation, chronic alcohol use, and coronary disease, and the American Society of Anesthesiologists (ASA) classification and American Spinal Cord Injury Association (ASIA) classification were recorded. The Injury Severity Score (ISS) was calculated manually based on the admission notes and imaging reports. Surgical factors including time to surgery, operative time, number of levels instrumented, and surgical approach were extracted from the EMR and validated against the intraoperative imaging to ensure accuracy. Postoperative adverse outcomes recorded included in-hospital mortality, any type of cerebrovascular accident (CVA), venous thromboembolic disease (DVT/PE), pressure injury, respiratory infection, urinary infection, and ventilator dependency.

Data analysis

All data analysis was performed using “R” (Ver 4.2.2 The Free Software Foundation Inc. Boston, MA). The Shapiro-Wilk test for normality was used, demonstrating that the data was not normally distributed. Continuous variables were expressed in terms of the median and interquartile range (IQR). Categorical variables were expressed as a percentage of the total sample number. Continuous variables were changed to categorical variables for the purpose of univariate analysis using the Chi-squared test.

Statistical model

A univariable selection method of logistic regression modeling was used. Models were constructed using all independent variables from the univariate analysis with p<0.20 [29,30]. Collinearity was assessed using the variance inflation factor (VIF), with VIF >10 used to define collinear variables. This threshold was chosen as it has been used in similar studies [9,31]. If collinearity was detected, the offending variable was removed from the analysis. This process was used to separately create regression models for each dependent variable. Independent variables were equally weighted within each model. The dataset was randomly split in a ratio of 70:30 for the purposes of creating training and testing subsets. These subsets were used to calculate the area under the curve (AUC) and McFadden’s R^2^ for each model.

## Results

Study population

A total of 92 patients met the inclusion criteria. The median patient age was 54.5 years (IQR: 32.5); 77 (82.2%) of the patients were male. The median ASA score was 3 (IQR: 2), with comorbidities including smoking (n=16, 17.2%), hypertension (n=26, 28.0%), diabetes (n=12, 13.0%), anticoagulation (n=4 4.3%), chronic alcoholism (n=6, 6.5%), and coronary disease (n=10 10.7%). The median ISS was 20 (IQR: 9). The median cervical level of injury was C4, and the median ASIA classification was ASIA C. The median number of levels instrumented was 3 (IQR: 3) with greater than 5 levels instrumented in 15 (16.3%) cases. The median surgical time was 171 minutes (IQR: 130).

Postoperative complications included CVA (n=1, 1.1%), DVT/PE (n=33, 35.5%), PU (n=5, 5.4%), respiratory infection (n=28, 30.1%), urinary tract infection (n=25, 26.9%), and ventilatory dependence (n=17, 18.3%). In-hospital mortality following surgery was 6.5% (n=6). Of these patients, the primary cause of death was respiratory failure in 83.3% (n=5).

The basic characteristics of the sample are presented in Table 3.

**Table 3:** Characteristics of the study participants (n=92) ASA: American Society of Anesthesiologists; ASIA: American Spinal Cord Injury Association; CVA: cerebrovascular accident; DVT/PE: deep vein thrombosis and/or pulmonary embolism; HTN: hypertension; IQR: interquartile range; ISS: Injury Severity Score; SD: standard deviation

Characteristics		
Demographics		
	Number of patients	92
	Age, years, median (IQR)	54.5 (32.5)
	Males, n (%)	77 (82.8)
Injury factors		
	ISS, median (IQR)	20.0 (9.0)
	Cervical level of injury, median (SD)	4.0 (1.0)
	Cervical 1-4 (high tetraplegia), n (%)	58 (63.0)
	Cervical 5-8 (low tetraplegia), n (%)	41 (37.0)
	ASIA classification, median	C
Surgery factors		
	Number of levels instrumented, median (SD)	3.0 (3.0)
	Greater than 5 levels instrumented, n (%)	15 (16.3)
	Surgery time, minutes, median (IQR)	171.0 (130.0)
Patient factors		
	ASA classification, median (IQR)	3 (2)
	Smoking, n (%)	16 (17.2)
	HTN, n (%)	26 (28.0)
	Diabetes, n (%)	12 (13.0)
	Anticoagulation, n (%)	4 (4.3)
	Chronic alcoholism, n (%)	6 (6.5)
	Coronary disease, n (%)	10 (10.7)
Complications		
	CVA, n (%)	1 (1.1)
	DVT/PE, n (%)	33 (35.5)
	Pressure ulcers, n (%)	5 (5.4)
	Respiratory infection, n (%)	28 (30.1)
	Urinary tract infection, n (%)	25 (26.9)
	Ventilator dependency, n (%)	17 (18.3)
	Postoperative in-hospital death, n (%)	6 (6.5)

Univariate analysis

In-hospital mortality was associated with age >65 years (Χ^2^=2.6, p=0.107), hypertension (Χ^2^=2.9, 0.091), anticoagulation (Χ^2^=6.6, p=0.01), chronic alcoholism (Χ^2^=3.6, p=0.058), coronary disease (Χ^2^=6.2, p=0.012), complete injury (Χ^2^=6.4, p=0.011), polytrauma (Χ^2^=2.7, p=0.099), greater than 5 levels of instrumentation (Χ^2^=3.02, p=0.0819), anterior instrumentation (Χ^2^=1.9, p=0.174), in-hospital thromboembolic disease (Χ^2^=2.1, p=0.145), and ventilator dependency (Χ^2^=13.6, p<0.001). Postoperative CVA was associated with chronic alcoholism (Χ^2^=3.1, p=0.076). Postoperative DVT/PE was associated with polytrauma (ISS >15) (Χ^2^=2.0, p=0.156). Postoperative pneumonia was associated with male gender (Χ^2^=3.5, p=0.06), age >65 years (Χ^2^=2.6, p=0.064), hypertension (Χ^2^=2.9, 0.085), complete injury (Χ^2^=2.6, 0.009), and polytrauma (Χ^2^=2.6, 0.002). Urinary tract infection was associated with high-level C-spine injury (C1-4) (Χ^2^=2.6, p=0.113). Ventilator dependence was associated with complete injuries (Χ^2^=19.6, p<0.001), and polytrauma (Χ^2^=12.1, p<0.001). These results are presented in Tables 4-10.

**Table 4:** Univariate analysis for dependent complication variable: in-hospital mortality Degrees of freedom=1; sample size=92 P-value <0.2 was the threshold for inclusion in multivariable logistic regression analysis DVT/PE: deep vein thrombosis and/or pulmonary embolism; ISS: Injury Severity Score; UTI: urinary tract infection

	Χ^2^ statistic	P-value
Patient factors		
Male gender	0.3	0.585
Age >65 years	2.6	0.107
Age >80 years	0.5	0.469
Smoking	0.3	0.611
hypertension	2.9	0.091
Diabetes	0.8	0.368
Anticoagulation	6.6	0.01
Alcoholism	3.6	0.058
Coronary disease	6.2	0.012
Injury factors		
Complete injury	6.4	0.011
High level (C1-C4)	0	1
Polytrauma (ISS >15)	2.7	0.099
Surgery factors		
Surgical time >120 minutes	0.01	0.901
Greater than 5 levels instrumented	3.02	0.0819
Anterior instrumentation	1.9	0.174
Posterior instrumentation	0.6	0.413
Anterior and posterior instrumentation	0	1
Complications		
In-hospital stroke	0	1
In-hospital DVT/PE	2.1	0.145
In-hospital pressure injury	0	1
In-hospital respiratory infection	0.4	0.536
In-hospital UTI	1.2	0.283
Ventilator dependency	13.6	<0.001

**Table 5:** Univariate analysis for dependent complication variable: CVA Degrees of freedom=1; sample size=92 P-value <0.2 was the threshold for inclusion in multivariable logistic regression analysis CVA: cerebrovascular accident; ISS: Injury Severity Score

	Χ^2^ statistic	P-value
Patient factors		
Male gender	0.8	0.359
Age >65 years	0.2	0.648
Age >80 years	0	1
Smoking	0	1
Hypertension	0.2	0.627
Diabetes	1.2	0.269
Anticoagulation	0	1
Alcoholism	3.1	0.076
Coronary disease	0	1
Injury factors		
Complete injury	0	1
High level (C1-C4)	0	1
Polytrauma (ISS >15)	0.04	0.841
Surgery factors		
Surgical time >120 minutes	0.2	0.605
Greater than 5 levels instrumented	0	1
Anterior instrumentation	0	1
Posterior instrumentation	0.02	0.877
Anterior and posterior instrumentation	0	1

**Table 6:** Univariate analysis for dependent complication variable: DVT/PE Degrees of freedom=1; sample size=92 P-value <0.2 was the threshold for inclusion in multivariable logistic regression analysis DVT/PE: deep vein thrombosis and/or pulmonary embolism; ISS: Injury Severity Score

	Χ^2^ statistic	P-value
Patient factors		
Male gender	0	1
Age >65 years	0.2	0.697
Age >80 years	0	1
Smoking	1.6	0.199
Hypertension	0	0.933
Diabetes	0.3	0.603
Anticoagulation	0.9	0.319
Alcoholism	0.1	0.759
Coronary disease	0.4	0.523
Injury factors		
Complete injury	0.8	0.386
High level (C1-C4)	0.6	0.445
Polytrauma (ISS >15)	9	0.002
Surgery factors		
Surgical time >120 minutes	0.5	0.473
Greater than 5 levels instrumented	0.4	0.51
Anterior instrumentation	1.4	0.228
Posterior Instrumentation	5.8	0.015
Anterior and posterior Instrumentation	1.1	0.283

**Table 7:** Univariate analysis for dependent complication variable: pressure injury Degrees of freedom=1; sample size=92 P-value <0.2 was the threshold for inclusion in multivariable logistic regression analysis ISS: Injury Severity Score

	Χ^2^ statistic	P-value
Patient factors		
Male gender	0.2	0.694
Age >65 years	0	1
Age >80 years	0	1
Smoking	0.2	0.653
Hypertension	0	1
Diabetes	0.04	0.835
Anticoagulation	0	1
Alcoholism	0.1	0.746
Coronary disease	0	1
Injury factors		
Complete injury	0	0.973
High level (C1-C4)	0	1
Polytrauma (ISS >15)	2	0.156
Surgical factors		
Surgical time >120 minutes	0	1
Greater than 5 levels instrumented	0	1
Anterior instrumentation	0	1
Posterior instrumentation	0.1	0.723
Anterior and posterior instrumentation	0.3	0.579

**Table 8:** Univariate analysis for dependent complication variable: respiratory infection Degrees of freedom=1; sample size=92 P-value <0.2 was the threshold for inclusion in multivariable logistic regression analysis ASIA: American Spinal Cord Injury Association; ISS: Injury Severity Score

	Χ^2^ statistic	P-value
Patient factors		
Male gender	3.5	0.06
Age >65 years	3.4	0.064
Age >80 years	0	1
Smoking	0.9	0.329
Hypertension	2.9	0.085
Diabetes	0.6	0.438
Anticoagulation	0.6	0.425
Alcoholism	0.1	0.764
Coronary disease	0.2	0.692
Injury factors		
Complete injury (ASIA A)	6.9	0.009
High level (C1-C4)	0.3	0.589
Polytrauma (ISS >15)	9.7	0.002
Surgical factors		
Surgical time >120 minutes	1.2	0.282
Greater than 5 levels instrumented	1.4	0.235
Anterior instrumentation	0	1
Posterior Instrumentation	0	1
Anterior and posterior instrumentation	0.3	0.559

**Table 9:** Univariate analysis for dependent complication variable: UTI Degrees of freedom=1; sample size=92 P-value <0.2 was the threshold for inclusion in multivariable logistic regression analysis ISS: Injury Severity Score; UTI: urinary tract infection

	Χ^2^ statistic	P-value
Patient factors		
Male gender	0.1	0.788
Age >65 years	0.4	0.549
Age >80 years	0.2	0.677
Smoking	0	1
Hypertension	0.1	0.821
Diabetes	0.3	0.596
Anticoagulation	0.2	0.635
Alcoholism	0	0.901
Coronary disease	0.8	0.359
Injury factors		
Complete injury	0.2	0.666
High level (C1-C4)	2.5	0.113
Polytrauma (ISS >15)	1.4	0.222
Surgery factors		
Surgical time >120 minutes	0.5	0.495
Greater than 5 levels instrumented	0.8	0.366
Anterior instrumentation	0.9	0.34
Posterior instrumentation	0.2	0.669
Anterior and posterior instrumentation	0.1	0.719

**Table 10:** Univariate analysis for dependent complication variable: ventilator dependency Degrees of freedom=1; sample size=92 P-value <0.2 was the threshold for inclusion in multivariable logistic regression analysis ISS: Injury Severity Score

	Χ^2^ statistic	P-value
Patient factors		
Male gender	0	0.843
Age >65 years	0.8	0.379
Age >80 years	0	1
Smoking	0.1	0.746
Hypertension	0	0.855
Diabetes	0	0.821
Anticoagulation	0	1
Alcoholism	0	1
Coronary disease	0	1
Injury factors		
Complete injury	19.6	<0.001
High level (C1-C4)	0.2	0.663
Polytrauma (ISS >15)	12.1	<0.001
Surgical factors		
Surgical time >120 minutes	0.005	0.942
Greater than 5 levels instrumented	0.3	0.596
Anterior instrumentation	0	0.896
Posterior instrumentation	0	0.873
Anterior and posterior instrumentation	0	0.906

Multivariable analysis

**Figure 3:**
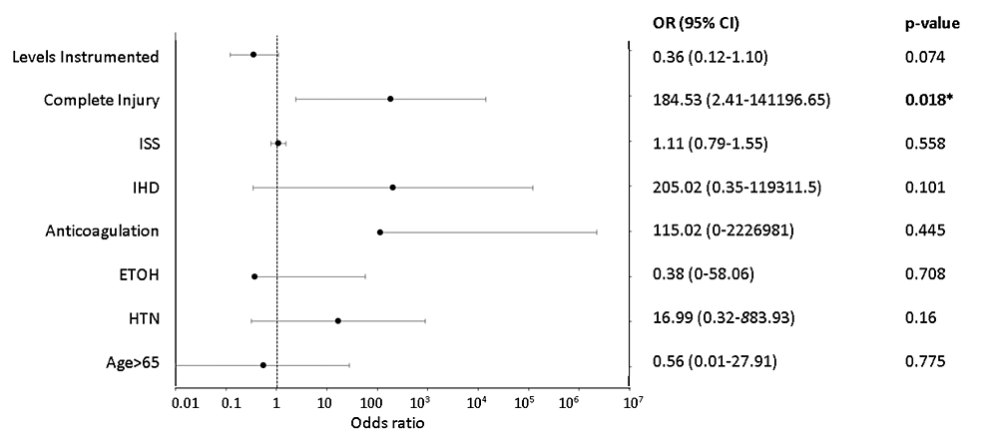
Plot of logistic regression model for in-hospital mortality *Significant at p<0.05 Formula: in-hospital mortality ~age >65 + HTN + ETOH + anticoagulation + IHD + complete injury + levels Instrumented CI: confidence interval; ETOH: chronic alcoholism; HTN: hypertension; IHD: ischemic heart disease; ISS: Injury Severity Score; OR: odds ratio

The independent variables used to create a regression model for in-hospital mortality were as follows: age >65 years, hypertension, alcoholism, anticoagulation, coronary disease, complete injury, ventilator dependency, and PE/DVT. The process of collinearity assessment and model selection is outlined in Table 11. The results of the chosen regression model for in-hospital mortality are depicted in Figure 3. In this model, complete injury was significantly associated with in-hospital mortality [odds ratio (OR): 184.53, 95% confidence interval (CI): 2.41-14106.65, p=0.018]. The AUC for this model was 0.9615 and R^2^=0.58.

**Table 11:** Collinearity analysis for dependent variable: in-hospital mortality ^⧫^Model chosen for multivariable analysis Variance inflation factors (VIF) for parameters of each model. Model variables were chosen from univariate analysis where p<0.2. VIF cut-off threshold was 10 ASIA: American Spinal Cord Injury Association classification; DVT/PE: deep vein thrombosis and/or pulmonary embolism; ETOH: chronic alcoholism; HTN: hypertension; IHD: ischemic heart disease; ISS: Injury Severity Score

	Age >65 years	HTN	ETOH	Anticoagulation	IHD	ISS	ASIA	Anterior	Levels instrumented	Ventilator dependency	DVT/PE
Age >65 years + HTN	1.4	1.4									
Age >65 years + HTN + ETOH	1.4	1.4	1.1								
Age >65 years + HTN + ETOH + IHD	1.6	1.4	1.2	1.3							
Age >65 years + HTN + ETOH + IHD + anticoagulation	1.9	1.6	1.2	1.2	1.3						
Age >65 years + HTN + ETOH + IHD + anticoagulation + ISS	1.9	1.7	1.2	1.3	1.4	1.1					
Age >65 years + HTN + ETOH + IHD + anticoagulation + ISS + ASIA	2.5	2	1.5	1	2.6	1.4	2.4				
Age >65 years + HTN + ETOH + IHD + anticoagulation + ISS + ASIA + anterior	2.1	2.3	1.8	1	1535.2	1.6	1.5	1535.2			
Age >65 years + HTN + ETOH + IHD + anticoagulation + ISS + ASIA + levels instrumented^⧫^	2.4	2.5	2.2	1.1	5.7	1.6	3.9	-	2.8		
Age >65 years + HTN + ETOH + IHD + anticoagulation + ISS + ASIA + levels instrumented + ventilator dependency	39.9	2.7	3.1	1.1	33.1	1.9	3.2	-	1	4.2	
Age >65 years + HTN + ETOH + IHD + anticoagulation + ISS + ASIA + levels instrumented + DVT/PE	72.4	13.8	1.4	1	69.2	1.01	26.7	-	1	-	4.3

## Discussion

Significant results

Our strongest finding pertaining to in-hospital mortality was its association with anticoagulation, coronary disease, complete injury, and ventilator dependency. The logistic regression model found complete injury to be significantly associated with postoperative in-hospital mortality. Studies have demonstrated a significant association between a high number of medical comorbidities and in-hospital mortality following SCI [10,19,32]. However, there is a paucity of evidence as to which specific chronic comorbidities contribute the most to postoperative complications in these patients. We have shown that in-hospital mortality risk after surgery for cervical SCI may be more strongly associated with cardiovascular comorbidities than diabetes and chronic alcohol abuse. We found that postoperative pneumonia and ventilator dependency was significantly associated with complete injury and polytrauma, as has been shown previously [33]. These findings highlight the vigilance required to prevent deterioration from respiratory decompensation in the ASIA A cohort of patients. Evidence supports providing specialized respiratory care for cervical SCI patients as a critical component of early management to avoid adverse outcomes [34].

Comparison of our findings with the literature

Table 12 provides a summary of previous studies investigating the risk factors for in-hospital mortality and complications among SCI patients using predictive models. The study population’s mean age of 51 years and its male predominance are consistent with similar studies [10,34-37]. The average level of injury was C4-5, which aligns with other studies [38-40]. However, the sample size of 92 was smaller when compared to larger studies in the literature with over 1000 patients [9,11,17,18,21]. Beck et al. investigated 706 patients with SCI in an Australian population and observed a median age of 50 years and an in-patient mortality of 15%, which is higher than our rate of 6.5%. However, their study was not limited to cervical SCI, and the inclusion criteria limited patients to those suffering from major trauma (ISS >12, ICU admission), which may account for their higher rate of in-hospital death. The majority of deaths in our study were secondary to respiratory complications, which is consistent with the literature [9-11]. Several studies have demonstrated minor differences in early complication rates between patients managed conservatively and those treated surgically [18,33], which may allow us to draw comparisons between our study cohort and nonoperatively managed patients.

**Table 12:** Summary of articles that investigate in-hospital mortality and complications with statistical relationships to various risk factors for SCI patients ASIA: American Spinal Cord Injury Association; GCS: Glasgow Coma Scale; ISS: Injury Severity Score; SCI: spinal cord injury; TBI: traumatic brain injury

Author, year, country	Sample	In-hospital mortality	The primary cause of death	Analysis technique: factors associated with in-hospital mortality
Shao et al. [9], 2011, China	N=1163, cervical SCI	9.4% (n=109)	Respiratory failure, 87.2% (n=95)	Logistic regression: -age -ASIA A -time to surgery -malnutrition -tracheotomy
Selassie et al. [17], 2012, USA	N=3389, all SCI	8.0% (n=272)	-	Cox proportional hazard regression model: -age -level of injury -2 or more comorbidities -concomitant injuries -pulmonary embolism
Durga et al. [33], 2010, India	N=101, postoperative, cervical SCI	17.8%	-	Logistic regression: -ASIA -haemodynamic instability -pulmonary infection
Chhabra et al. [10], 2019, India	N=758, all SCI	10% (n=79)	Respiratory complications including pulmonary embolism and pneumonia (42%)	Cox proportional hazard regression: -age -low tetraplegia -ventilator use -associated injuries -cardiovascular complication
Inglis et al. [19], 2020, Canada	N=826, postoperative all SCI, age over 65 years	9.5% (n=78)	-	Logistical regression: -age -ASIA A -Charlson Comorbidity Index -ventilation dependence
Gong et al. [11], 2022, China	N=3176, all SCI	0.7% (n=23)	Respiratory complication, 73.9% (n=17)	Logistic regression: -cervical level -abdominal visceral injury -ASIA classification -surgery
Blex et al. [32], 2022, Germany	N=321, all SCI	6.2% (n=20)	Multiorgan failure, 40.0% (n=8)	Condition inference tree analysis: -age -Charlson Comorbidity Index -kidney or liver impairment
Shibahashi et al. [21], 2019, Japan	N=8069, all SCI	5.6%	-	Logistic regression: -age -male -GCS -hypotension on arrival -bradycardia on arrival -severe head injury -ISS -ASIA classification
Varma et al. [18], 2010, USA	N=1995, all SCI	12.6% (n=251)	-	Logistic regression: -age -male -ISS >15 -TBI -1 or more comorbidities -ASIA classification
Jackson et al. [20], 2005, USA	N=458, postoperative, cervical SCI	3.9%	-	-Age >65 years -ASIA A
Beck et al. [41], 2019, Australia	N=706, all SCI	15% (n=104)	-	Χ^2 ^test: -age

Beck et al. showed that Australia is witnessing an increase in the number of older adults with traumatic SCI [41], which is consistent with other studies [36,42]. These findings exceed the rate of geriatric population growth and reflect an increasing rate of trauma from low falls in this age group [43,44]. Most studies modeling risk factors for complications following SCI report increasing age as significantly associated with in-hospital mortality [9,17-20,32,41]. Inglis et al. specifically investigated traumatic SCI in a geriatric population (n=1340) and determined that the odds of dying 50 days post-surgery are six times higher for patients aged ≥77 years versus those aged 65-76 years [19]. Our study did not find a strong association between age and postoperative complications. Some studies in the literature also demonstrate an insignificant relationship between age and postoperative mortality following cervical SCI surgery [33]. Wilson et al. investigated a group of geriatric (age >65 years) cervical SCI patients managed surgically [45]. Mortality at 90 days was significantly associated with complete injury; however, there was no significant association between mortality and age subgroups. A possible explanation for this inconsistency could be selection bias, as older patients are more likely to be conservatively managed without surgery, which avoided their inclusion in the study.

Strengths, limitations, and future research

The main strength of this study lies in the reliability of the dataset, which was rigorously extracted using multiple ICD codes and validated using multiple sources [46]. Evidence suggests significant inaccuracies inherent in hospital coding data [47-50] and underreporting of adult variants of SCI without radiological abnormality (SCIWORA) [51,52]. The data collection methodology employed in this study used multiple validation points between coding, EMR documentation, and medical imaging in an attempt to reduce information bias.

The relatively small sample size of this project likely contributed to statistical errors. A power analysis during the study planning phase would have been useful [53], as it is generally accepted that a larger sample size can improve the validity of a model by reducing the confounding errors caused by multicollinearity [54]. A larger sample size could be derived from local and state-wide trauma registries. A more detailed dataset (including time of death) would allow for Kaplan-Meier survival curves to be drawn and a Cox proportional hazard model to be constructed, as performed in similar studies [10,17]. Logistic regression models may be less precise than a Cox proportional hazard model and may have difficulty in separating the effects of inter-related, time-dependent covariates [55,56]. This model could then be validated on an external, compatible dataset to improve external validity, as has been performed in similar studies [33,57].

This study was limited to a single centre and did not include patients admitted under the neurosurgical team. Additionally, the study design was retrospective and conducted by a single author, reducing overall reliability and allowing for potential bias. As with most retrospective, non-randomised surgical studies, selection bias towards healthier, operative candidates is an inherent factor [58]. The scope of this study did not include nonoperative patients, a group more likely to be palliated secondary to old age and comorbidities, precluding surgical management. Additionally, the scope of this study was limited to patients admitted under orthopaedics. The neurosurgical team admits approximately 30% of acute SCI patients at RNSH and there is evidence to suggest that there is a lack of consensus between neurosurgeons and orthopaedic surgeons on the value of surgical intervention after SCI [59], which may have influenced the results. The grouping of venous thromboembolic disease into PE + DVT may have confounded the association of PE with mortality risk, which has been established in the literature [17]. 

The future of this type of research will involve the development of machine-learning algorithms to better understand non-linear associations between variables and possibly model mortality risk with greater precision. One recent study has suggested the superiority of a machine-learning-derived tool, the SCI Risk Score (SCIRS), which can predict in-hospital and one-year mortality using the following variables: age, neurological level and completeness of injury, fracture classification, and Abbreviated Injury Scale scores. This tool has been validated in a separate cohort and demonstrated superior AUC and sensitivity than ISS [60].

## Conclusions

To improve surgical outcomes for patients with traumatic cervical SCI, a concerted effort must be made to prevent postoperative complications. This study investigated the patient, injury, and surgical factors contributing to in-hospital mortality and postoperative complications following surgery for traumatic SCI. Cardiovascular comorbidities including anticoagulation and coronary disease appear to present significant patient risk factors contributing to in-hospital mortality. A logistic regression analysis found complete neurological injury to be significant in predicting in-hospital mortality. Patient age appears to insignificantly influence postoperative complication rates; however, this finding may have been influenced by selection bias. Postoperative respiratory complications, especially in the setting of complete injury and ventilator dependency, are particularly devastating. The findings of this study endorse the existing literature advocating for vigilant respiratory management in these fragile patients.

## References

[1] New PW, Baxter D, Farry A, Noonan VK (2015). Estimating the incidence and prevalence of traumatic spinal cord injury in Australia. Arch Phys Med Rehabil.

[2] O'Connor P (2002). Incidence and patterns of spinal cord injury in Australia. Accid Anal Prev.

[3] National Spinal Cord Injury Statistical Center (2013). Spinal cord injury facts and figures at a glance. J Spinal Cord Med.

[4] Alizadeh A, Dyck SM, Karimi-Abdolrezaee S (2019). Traumatic spinal cord injury: an overview of pathophysiology, models and acute injury mechanisms. Front Neurol.

[5] von Leden RE, Yauger YJ, Khayrullina G, Byrnes KR (2017). Central nervous system injury and nicotinamide adenine dinucleotide phosphate oxidase: oxidative stress and therapeutic targets. J Neurotrauma.

[6] Oyinbo CA (2011). Secondary injury mechanisms in traumatic spinal cord injury: a nugget of this multiply cascade. Acta Neurobiol Exp (Wars).

[7] Dumont RJ, Okonkwo DO, Verma S, Hurlbert RJ, Boulos PT, Ellegala DB, Dumont AS (2001). Acute spinal cord injury, part I: pathophysiologic mechanisms. Clin Neuropharmacol.

[8] Liebscher T, Ludwig J, Lübstorf T (2022). Cervical spine injuries with acute traumatic spinal cord injury: spinal surgery adverse events and their association with neurological and functional outcome. Spine (Phila Pa 1976).

[9] Shao J, Zhu W, Chen X (2011). Factors associated with early mortality after cervical spinal cord injury. J Spinal Cord Med.

[10] Chhabra HS, Sharawat R, Vishwakarma G (2022). In-hospital mortality in people with complete acute traumatic spinal cord injury at a tertiary care center in India-a retrospective analysis. Spinal Cord.

[11] Gong Y, Du J, Hao D (2022). A new scale for predicting the risk of in-hospital mortality in patients with traumatic spinal cord injury. Front Neurol.

[12] Casha S, Christie S (2011). A systematic review of intensive cardiopulmonary management after spinal cord injury. J Neurotrauma.

[13] Alabed S, de Heredia LL, Naidoo A, Belci M, Hughes RJ, Meagher TM (2015). Incidence of pulmonary embolism after the first 3 months of spinal cord injury. Spinal Cord.

[14] Cardenas DD, Hoffman JM, Kirshblum S, McKinley W (2004). Etiology and incidence of rehospitalization after traumatic spinal cord injury: a multicenter analysis. Arch Phys Med Rehabil.

[15] Johnson RL, Gerhart KA, McCray J, Menconi JC, Whiteneck GG (1998). Secondary conditions following spinal cord injury in a population-based sample. Spinal Cord.

[16] Gélis A, Dupeyron A, Legros P, Benaïm C, Pelissier J, Fattal C (2009). Pressure ulcer risk factors in persons with SCI: Part I: acute and rehabilitation stages. Spinal Cord.

[17] Selassie AW, Varma A, Saunders LL, Welldaregay W (2013). Determinants of in-hospital death after acute spinal cord injury: a population-based study. Spinal Cord.

[18] Varma A, Hill EG, Nicholas J, Selassie A (2010). Predictors of early mortality after traumatic spinal cord injury: a population-based study. Spine (Phila Pa 1976).

[19] Inglis T, Banaszek D, Rivers CS (2020). In-hospital mortality for the elderly with acute traumatic spinal cord injury. J Neurotrauma.

[20] Jackson AP, Haak MH, Khan N, Meyer PR (2005). Cervical spine injuries in the elderly: acute postoperative mortality. Spine (Phila Pa 1976).

[21] Shibahashi K, Nishida M, Okura Y, Hamabe Y (2019). Epidemiological state, predictors of early mortality, and predictive models for traumatic spinal cord injury: a multicenter nationwide cohort study. Spine (Phila Pa 1976).

[22] Badhiwala JH, Wilson JR, Witiw CD (2021). The influence of timing of surgical decompression for acute spinal cord injury: a pooled analysis of individual patient data. Lancet Neurol.

[23] Papadopoulos SM, Selden NR, Quint DJ, Patel N, Gillespie B, Grube S (2002). Immediate spinal cord decompression for cervical spinal cord injury: feasibility and outcome. J Trauma.

[24] Phan K, Kim JS, Kim JH, Somani S, Di'Capua J, Dowdell JE, Cho SK (2017). Anesthesia duration as an independent risk factor for early postoperative complications in adults undergoing elective ACDF. Global Spine J.

[25] Brodke DS, Anderson PA, Newell DW, Grady MS, Chapman JR (2003). Comparison of anterior and posterior approaches in cervical spinal cord injuries. J Spinal Disord Tech.

[26] Ren C, Qin R, Wang P, Wang P (2020). Comparison of anterior and posterior approaches for treatment of traumatic cervical dislocation combined with spinal cord injury: minimum 10-year follow-up. Sci Rep.

[27] (2022). Australian Bureau of Statistics: Snapshot of New South Wales. Australian Bureau of Statistics: Canberra, ABS.

[28] Australian Institute of Health and Welfare (2022). Australian Institute of Health and Welfare: Australian Family of Health and Related Classifications - ICD-10-AM. 2022.

[29] Mickey RM, Greenland S (1989). The impact of confounder selection criteria on effect estimation. Am J Epidemiol.

[30] Chowdhury MZ, Turin TC (2020). Variable selection strategies and its importance in clinical prediction modelling. Fam Med Community Health.

[31] Kim JH (2019). Multicollinearity and misleading statistical results. Korean J Anesthesiol.

[32] Blex C, Kreutzträger M, Ludwig J (2022). Baseline predictors of in-hospital mortality after acute traumatic spinal cord injury: data from a level I trauma center. Sci Rep.

[33] Durga P, Sahu BP, Mantha S, Ramachandran G (2010). Development and validation of predictors of respiratory insufficiency and mortality scores: simple bedside additive scores for prediction of ventilation and in-hospital mortality in acute cervical spine injury. Anesth Analg.

[34] Chamberlain JD, Meier S, Mader L, von Groote PM, Brinkhof MW (2015). Mortality and longevity after a spinal cord injury: systematic review and meta-analysis. Neuroepidemiology.

[35] Cao Y, Krause JS, DiPiro N (2013). Risk factors for mortality after spinal cord injury in the USA. Spinal Cord.

[36] DeVivo MJ, Krause JS, Lammertse DP (1999). Recent trends in mortality and causes of death among persons with spinal cord injury. Arch Phys Med Rehabil.

[37] Krause JS, Saunders LL, Zhai Y (2012). Stability of predictors of mortality after spinal cord injury. Spinal Cord.

[38] Brolin K, von Holst H (2002). Cervical injuries in Sweden, a national survey of patient data from 1987 to 1999. Inj Control Saf Promot.

[39] Pickett GE, Campos-Benitez M, Keller JL, Duggal N (2006). Epidemiology of traumatic spinal cord injury in Canada. Spine (Phila Pa 1976).

[40] Solagberu BA (2002). Spinal cord injuries in Ilorin, Nigeria. West Afr J Med.

[41] Beck B, Cameron PA, Braaf S (2019). Traumatic spinal cord injury in Victoria, 2007-2016. Med J Aust.

[42] Ahoniemi E, Alaranta H, Hokkinen EM, Valtonen K, Kautiainen H (2008). Incidence of traumatic spinal cord injuries in Finland over a 30-year period. Spinal Cord.

[43] Kehoe A, Smith JE, Edwards A, Yates D, Lecky F (2015). The changing face of major trauma in the UK. Emerg Med J.

[44] Beck B, Cameron P, Lowthian J, Fitzgerald M, Judson R, Gabbe BJ (2018). Major trauma in older persons. BJS Open.

[45] Wilson KV, McDonnell JM, O'Malley S (2022). Morbidity and mortality of traumatic cervical spinal cord injuries in a geriatric cohort. Ir J Med Sci.

[46] Hagen EM, Rekand T, Gilhus NE, Gronning M (2009). Diagnostic coding accuracy for traumatic spinal cord injuries. Spinal Cord.

[47] Weller CD, Turnour L, Connelly E, Banaszak-Holl J, Team V (2022). Clinical coders' perspectives on pressure injury coding in acute care services in Victoria, Australia. Front Public Health.

[48] Liu S, Kim D, Penfold S, Doric A (2022). Clinical documentation requirements for the accurate coding of hospital-acquired urinary tract infections in Australia. Aust Health Rev.

[49] MacIntyre CR, Ackland MJ, Chandraraj EJ, Pilla JE (1997). Accuracy of ICD-9-CM codes in hospital morbidity data, Victoria: implications for public health research. Aust N Z J Public Health.

[50] MacIntyre CR, Ackland MJ, Chandraraj EJ (1997). Accuracy of injury coding in Victorian hospital morbidity data. Aust N Z J Public Health.

[51] Boese CK, Lechler P (2013). Spinal cord injury without radiologic abnormalities in adults: a systematic review. J Trauma Acute Care Surg.

[52] Kasimatis GB, Panagiotopoulos E, Megas P, Matzaroglou C, Gliatis J, Tyllianakis M, Lambiris E (2008). The adult spinal cord injury without radiographic abnormalities syndrome: magnetic resonance imaging and clinical findings in adults with spinal cord injuries having normal radiographs and computed tomography studies. J Trauma.

[53] Olvera Astivia OL, Gadermann A, Guhn M (2019). The relationship between statistical power and predictor distribution in multilevel logistic regression: a simulation-based approach. BMC Med Res Methodol.

[54] Vatcheva KP, Lee M, McCormick JB, Rahbar MH (2016). Multicollinearity in regression analyses conducted in epidemiologic studies. Epidemiology (Sunnyvale).

[55] Leffondré K, Abrahamowicz M, Siemiatycki J (2003). Evaluation of Cox's model and logistic regression for matched case-control data with time-dependent covariates: a simulation study. Stat Med.

[56] Callas PW, Pastides H, Hosmer DW (1998). Empirical comparisons of proportional hazards, poisson, and logistic regression modeling of occupational cohort data. Am J Ind Med.

[57] Royston P, Altman DG (2013). External validation of a Cox prognostic model: principles and methods. BMC Med Res Methodol.

[58] Paradis C (2008). Bias in surgical research. Ann Surg.

[59] Hussain M, Nasir S, Moed A, Murtaza G (2011). Variations in practice patterns among neurosurgeons and orthopaedic surgeons in the management of spinal disorders. Asian Spine J.

[60] Fallah N, Noonan VK, Waheed Z (2022). Development of a machine learning algorithm for predicting in-hospital and 1-year mortality after traumatic spinal cord injury. Spine J.

